# Assessing the physical healthcare gap among patients with severe mental illness: large real-world investigation from Italy

**DOI:** 10.1192/bjo.2021.998

**Published:** 2021-09-09

**Authors:** Giovanni Corrao, Matteo Monzio Compagnoni, Valeria Valsassina, Antonio Lora

**Affiliations:** National Centre for Healthcare Research and Pharmacoepidemiology, University of Milano-Bicocca, Italy; and Unit of Biostatistics, Epidemiology and Public Health, Department of Statistics and Quantitative Methods, University of Milano-Bicocca, Italy; National Centre for Healthcare Research and Pharmacoepidemiology, University of Milano-Bicocca, Italy; and Unit of Biostatistics, Epidemiology and Public Health, Department of Statistics and Quantitative Methods, University of Milano-Bicocca, Italy; National Centre for Healthcare Research and Pharmacoepidemiology, University of Milano-Bicocca, Italy; and Unit of Biostatistics, Epidemiology and Public Health, Department of Statistics and Quantitative Methods, University of Milano-Bicocca, Italy; National Centre for Healthcare Research and Pharmacoepidemiology, University of Milano-Bicocca, Italy; and Department of Mental Health and Addiction Services, Azienda Socio Sanitaria Territoriale di Lecco, Italy

**Keywords:** Mental healthcare, physical healthcare gap, drug adherence, severe mental illness, healthcare utilisation database

## Abstract

**Background:**

One critical barrier to the uptake of mental health programmes is the so-called physical healthcare gap, a concern raised by the unattended physical comorbidity and early mortality of persons with severe mental illness.

**Aims:**

To evaluate the extension of physical healthcare gap among persons with severe mental illness under chronic drug therapies.

**Method:**

A population-based cohort study was carried out, using Lombardy healthcare utilisation databases. Prevalent patients treated with blood pressure-, lipid- or glucose-lowering agents were identified in January 2017. Among these, those who were receiving care for depression, schizophrenia, bipolar disorder or personality disorder formed the study cohort. A reference cohort was randomly selected from prevalent patients treated with chronic therapies without signs of severe mental disorders, to be matched with study cohort members for gender, age and number of previous contacts with the National Health System. One-year adherence to healthcare was measured through the proportion of days covered (drug adherence), and exposure to selected recommendations (clinical control adherence).

**Results:**

The 55 162 patients with severe mental illness were less likely to have high adherence to blood pressure-lowering, lipid-lowering or antidiabetic agents than the reference cohort by −24% (95% CI −26 to −22%), −10% (95% CI −14 to −6%) and −25% (95% CI −29 to −21%), respectively. The 9250 patients with diabetes and severe mental illness had −18% (95% CI −22% to −13%) reduced likelihood to meet recommendations for the clinical management of diabetes, compared with the reference cohort.

**Conclusions:**

Adherence to chronic drug therapies was sensibly worse among patients living with mental illness than those without signs of mental disorders.

Mental health epidemiologists and scholars of mental healthcare services are moving beyond traditional measures of incidence and prevalence to include treatment gap^1,2^ and assessment of unmet needs in psychiatry.^3,4^ A main issue in this field includes the physical healthcare gap, a concern raised by the relatively frequent but highly unattended physical comorbidity^5^ and early mortality of persons with severe mental illness.^6,7^

Patients with chronic physical conditions may be non-adherent to recommended care for several reasons, including their disbelief in the efficacy of treatment,^8^ the presence of barriers such as adverse effects,^9,10^ and lack of help and support from family members^11^ or health professionals. In addition, it is noteworthy that non-somatic diseases, like mental health disorders, might also affect patients’ ability or willingness to adhere to recommended treatments. Even when people receive care from mental health services, mental health professionals may not give adequate attention to the physical assessment of patients treated for psychiatric disorders.^12^ Indeed, having both a physical and mental health condition still results in more complicated treatments and poorer outcomes than having either problem alone.^13^

Assessing the extent to which non-adherence to recommended treatments for chronic somatic diseases might be a potentially avoidable concomitant effect of a non-somatic treatable condition (i.e. mental disorders) may be an important first step in improving patient adherence, the therapeutic alliance between physicians and patients, the outcomes of medical treatment^14,15^ and ultimately, to provide further evidence to support the need for integrating physical and mental health in public health policies.

## Aims of the study

The aim of the present study was to assess the extension of physical healthcare gap among persons with severe mental disorders under chronic drug therapy, evaluating the association between exposure to mental disorders and adherence to recommended healthcare for chronic somatic diseases. We identified three large, regional population-based cohorts of patients who were under chronic pharmacological therapy with blood pressure-lowering, lipid-lowering or glucose-lowering agents, some of whom had severe mental disorders, to investigate whether and to what extent mental illness affected the short-term adherence to recommended healthcare.

## Method

### Data sources

The study was based on the computerised healthcare utilisation (HCU) databases of Lombardy, an Italian northern region accounting for almost 10 million inhabitants (about 16% of the national population). In Italy, all citizens have equal access to healthcare provided by the National Health Service (NHS). Its management in Lombardy is associated with an automated system of HCU databases, which include a variety of information on the beneficiaries of the regional health service (virtually all residents in the region), such as diagnosis at discharge from public or private hospitals, out-patient drug prescriptions, specialist visits and diagnostic examinations provided fully or partly free of charge, by the NHS. In addition, a specific automated system concerning mental healthcare gathers data from regional Departments of Mental Health (DMHs) accredited by the NHS (i.e. the so-called ‘Italian Mental Health Information System’). This system provides demographic information and diagnostic and therapeutic codes for patients receiving specialist mental healthcare by the regional DMH facilities. These various types of data can be interconnected through a record-linkage procedure, since a unique individual identification code is used among all databases for each NHS beneficiary, to trace the complete healthcare pathway of each resident. To preserve privacy, each identification code is automatically anonymised, which can only be reversed by the regional authority upon request from judicial authorities. Further details on HCU database use in the field of mental healthcare have been reported elsewhere.^12,16^ Diagnostic and drug therapy codes used for drawing records and fields from the considered databases are reported in Supplementary Table 1 available at https://doi.org/10.1192/bjo.2021.998.

### Selecting the study and reference cohorts

Beneficiaries of the NHS who, on 1 January 2017 (index date), were aged 18 years or older, were resident in Lombardy for at least 2 years and were receiving treatment with blood pressure-lowering, lipid-lowering or antidiabetic agents, were identified and included as three separated groups. An individual was considered to be receiving treatment with a given drug therapy if, during the 2-year period before the index date (i.e. 2015–2016), they had received at least three consecutive dispensations of that drug therapy. Drug dispensations were considered consecutive (uninterrupted) if the timespan between the coverage end of one prescription and the beginning of the following prescription was 60 days or shorter, being the defined daily dose metrics assumed for calculating drug coverage. It is noteworthy that the three groups of prevalent patients treated with blood pressure-lowering, lipid-lowering or antidiabetic agents were not independent, since a patient on therapy with two (or three) of the considered medications was included in the corresponding two (or three) groups. Patients who died or moved to another region or country within 1 year of the index date were excluded from the groups (i.e. at least 1 year of observation was required for every participant).

Within each of the considered groups, patients diagnosed with a severe mental disorder who were receiving care from a mental health service at the index date were identified and considered as belonging to the study cohort. Patients were defined as receiving care when they had an active record in the Mental Health Information System of having received a diagnosis of depression, schizophrenia, bipolar disorder or personality disorder at any time before the index date, and were still receiving care for the disorder at the index date.

A reference cohort suitable to be used as comparator for the study cohort was generated. Patients who were eligible to be selected as comparators were those who belonged to the groups being treated with a given drug therapy (the same groups that generated the study cohorts), but had no mental health service provision recorded in the Mental Health Information System. For each study cohort member, up to three eligible comparators were randomly selected to be matched for gender, age at index date (±1 year) and number of contacts with the NHS (i.e. drug dispensations, hospital admissions, out-patient visits and procedures) in the 2 years before the index date.

Both study and reference cohort members were followed from the index date until 1 year after the index date (end-point of follow-up).

### Assessing adherence to healthcare

Starting from the index date, all medications dispensed during the following year to patients belonging to study and reference cohorts were recorded. The duration of each prescription was calculated by dividing the total amount of the drug prescribed by the defined daily dose. Adherence to drug therapy was measured by the ratio between the cumulative number of days in which the drug was available and days of the overall follow-up (proportion of days covered; PDC).^17,18^ Adherence was categorised according with PDC value as very low (PDC ≤ 25%), low (26–50%), intermediate (51–74%) and high (≥75%).

Because we have no information about in-hospital dispensed drugs, therapeutic regimens observed before hospital admission was assumed as continuously administered during the hospital stay, taking into account the so-called ‘immeasurable time bias’.^19^

Out-patient clinical controls, including assessments of lipid profile (total and HDL cholesterol and triglycerides), serum creatinine, glycated haemoglobin, urine albumin excretion and dilated eye examinations dispensed to cohort members on drug therapy with antidiabetic agents, were identified. A patient was considered as adherent to these recommendations when they had at least two glycated haemoglobin assays and at least one of the other evaluations, annually.^20,21^ Overall, a high adherence to recommended clinical controls was considered to be reached for those patients who underwent all, or almost all, recommendations (i.e. when at least four of the five controls were performed during the first year after the index date).^22^

### Additional measurements

Baseline characteristics of study and reference cohort members included comorbidities and cotreatments (antithrombotic, antiarrhythmics and antineoplastic agents, digitalis, nitrates, nonsteroidal anti-inflammatory drugs (NSAIDs) and drugs for pulmonary diseases). Comorbidities was identified from in-patient diagnoses and out-patient drug treatments experienced within the 2 years before the index date. In addition, patients were categorised according to the Multisource Comorbidity Score (MCS), a new index of patients’ clinical status derived from in-patient diagnostic information and out-patient drug prescriptions, provided by the regional Italian data and validated for outcome prediction.^23^

### Data analysis

Baseline characteristics of study and reference cohort members were compared by means of absolute standardised differences (we considered an absolute standardised difference of <0.10 as negligible^24^). Comparisons regarding adherence to healthcare recommendations were made by means of chi-square test, or its version for the trend, where proper (*P* < 0.05 was considered to be significant).

Multivariable conditional logistic regression was fitted for modelling the odds ratio and 95% confidence interval, for the association between the exposure and the outcome(s) of interest. Cardinal exposure was the condition of having a diagnosis of mental disorder or not, i.e. to belong to the study or reference cohort, respectively. A patient was considered as having experienced the outcome when, during the follow-up period, at least 75% of the drug was available (i.e. the patient had a high drug adherence, PDC ≥ 75%) or at least four of the five controls were performed (i.e. high clinical control adherence). Adjustments for the covariates listed in the above ‘Additional measurements’ section were performed. Stratified analyses were performed according to predefined stratification variables (diagnostic categories of mental disorders, gender, age classes and categories of MCS). In other terms, adjusted conditional logistic regression was fitted as described, separately for each strata of the stratification variables. Between-strata homogeneity of odds ratios (type of mental disorder and gender) and along-strata trend (categories of age and MCS) were tested for model estimates. The hypothesis of homogeneity among strata was tested with the chi-square test, whereas trends in odds ratio were tested with the statistical significance of the regression coefficient of the recoded variable, obtained by scoring the corresponding categories of adherence. The regression coefficients of the outcome risk trends between strata were compared with the *z*-test.

The exposure → outcome association was expressed by (odds ratio−1)×100, a percentage variation of the likelihood of high adherence to recommendations among patients with a severe mental disorder versus patients without a severe mental disorder. Statistical evidence of reduced adherence was assumed when a negative value of the quantity (lower bound of the confidence interval at 95% of the (odds ratio−1)×100) was obtained. Conversely, significant increased adherence was assumed when a positive value of the quantity (upper bound of the confidence interval at 95% of the (odds ratio−1)×100) was obtained.

To verify the robustness of our findings, we adopted different ways for the categorisation of drug and controls adherence, i.e. a patient was considered having experienced the outcome when at least 70 or 80% of the follow-up period was covered by the drug therapy (instead of the 75% cut-off adopted for the main analysis), or at least three of the five controls were performed (instead of the four controls adopted in the main analysis).

The software SAS (version 9.4 for Windows; SAS Institute, North Carolina, USA) was used to perform all analyses.

### Ethical issues

The Ethical Committee of the University of Milano-Bicocca evaluated the protocol (protocol number 497, year 2019) and established that the study was exempt from informed consent (according to General Authorization for the Processing of Personal Data for Scientific Research Purposes issued by the Italian Privacy Authority on 10 August 2018; https://www.gpdp.it/web/guest/home/docweb/-/docweb-display/docweb/9124510); provides sufficient guarantees of individual records anonymity and was designed according to quality standards of good practice of observational research based on secondary data.

## Results

### Patients

**Fig. 1:**
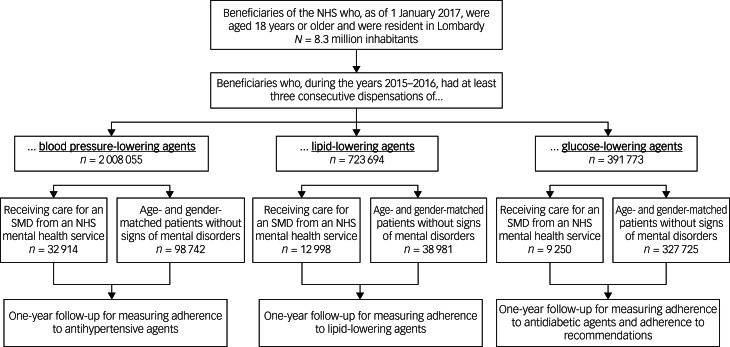
Flow chart showing criteria for eligibility of prevalent patients treated with blood pressure-, lipid- and glucose-lowering agents and, within each category of drug therapy, patients with and without mental disorders. NHS, National Health Service; SMD, severe mental disorders (depression, schizophrenia, bipolar disorder and personality disorders).

The process of cohort selection is shown in Fig. 1. Among the 8.3 million inhabitants from Lombardy aged 18 years or older on 1 January 2017, 2 008 055 (24%), 723 694 (9%) and 391 773 (5%) were on treatment with blood pressure-, lipid- and glucose-lowering agents, respectively. Around 2% were receiving care at the facilities of regional DMHs, forming the study cohorts of 32 914, 12 998 and 9250 prevalent patients treated with blood pressure-, lipid- and glucose-lowering agents with a diagnosis of a severe mental disorder. Patients without a severe mental disorder were 1:3 matched, forming the reference cohort.

The baseline characteristics of study and reference cohort members are compared in Table 1. The mean age (s.d.) was around 65 (s.d. 12) years, and women were systematically more represented. Although co-medications were essentially similar in study and reference cohort members (with a few exceptions; for example, NSAIDs were more frequently prescribed among study cohort members than comparators), patients with mental disorders had a worse clinical profile on average than reference cohort members.

**Table 1:** Baseline characteristics of study and reference cohort members receiving treatment with antihypertensives, statins or antidiabetics in Lombardy, Italy in the period 2016–2017

	Prevalent patients treated with:								
	Blood pressure-lowering agents			Lipid-lowering agents			Glucose-lowering agents		
	Study cohort, *n* = 32 914	Reference cohort, *n* = 98 742	Absolute standardised difference^a^	Study cohort, *n* = 12 998	Reference cohort, *n* = 38 981	Absolute standardised difference^a^	Study cohort, *n* = 9250	Reference cohort, *n* = 27 725	Absolute standardised difference^a^
Demographics									
Men	40.2%	40.2%	Matching variable	42.0%	42.0%	Matching variable	46.6%	46.6%	Matching variable
Age (years)									
18–40	2.4%	2.4%	Matching variable	1.2%	1.2%	Matching variable	2.6%	2.6%	Matching variable
41–65	47.9%	47.9%		46.5%	46.5%		50.7%	50.7%	
>65	49.7%	49.7%		52.3%	52.3%		46.7%	46.7%	
Co-medications									
Glucose-lowering agents	21.2%	16.5%	12.2%	32.4%	27.6%	10.6%	−	−	−
Blood pressure-lowering agents	−	−	−	74.2%	79.1%	11.8%	72.0%	76.5%	10.4%
Lipid-lowering agents	33.5%	33.6%	0.2%	−	−	−	49.2%	53.2%	7.9%
Antiarrhythmics	3.6%	4.4%	4.4%	4.0%	4.8%	3.9%	2.5%	2.8%	1.6%
Antithrombotic agents	42.5%	37.2%	10.8%	53.4%	50.9%	5.0%	50.2%	41.7%	7.1%
Digitalis	1.3%	1.1%	1.9%	0.9%	0.8%	11.7%	1.1%	1.1%	0.5%
Nitrates	4.4%	3.9%	2.4%	7.3%	7.3%	0.2%	4.9%	4.9%	0.1%
Drugs for COPD	24.0%	21.4%	6.1%	23.6%	21.9%	4.1%	22.4%	21.0%	3.4%
Antineoplastic agents	1.6%	1.9%	2.8%	1.3%	1.6%	1.8%	1.4%	1.7%	2.1%
NSAIDs	46.3%	41.0%	10.7%	47.6%	42.1%	11.2%	45.7%	43.8%	3.8%
Clinical profile^b^									
Good	67.5%	77.3%	50.5%	65.1%	72.7%	18.2%	56.4%	65.1%	19.3%
Intermediate	20.4%	15.0%		22.7%	18.7%		26.8%	22.9%	
Poor	12.1%	7.7%		12.3%	8.6%		16.8%	12.0%	

COPD, chronic obstructive pulmonary disease; NSAIDs, non-steroidal anti-inflammatory drugs.

a. Absolute standardised difference comparing patients with mental disorders and matched reference cohort members.

b. According to the Multisource Comorbidity Score.

### Adherence to healthcare

**Fig. 2:**
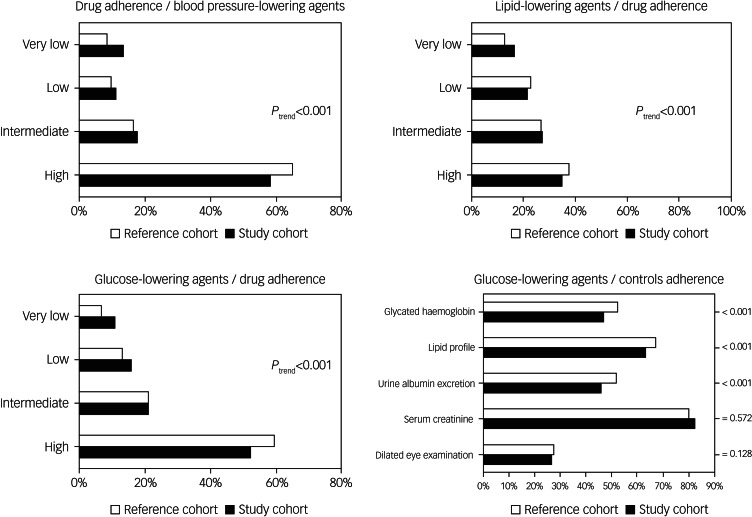
Distribution of reference and study cohort members according to the categories of adherence to drug therapy and clinical controls. Adherence to each pharmacological therapy is categorised as very low (≤25%), low (26–50%), intermediate (51–74%) and high (≥75%) proportion of days covered by drug prescriptions.

**Fig. 3:**
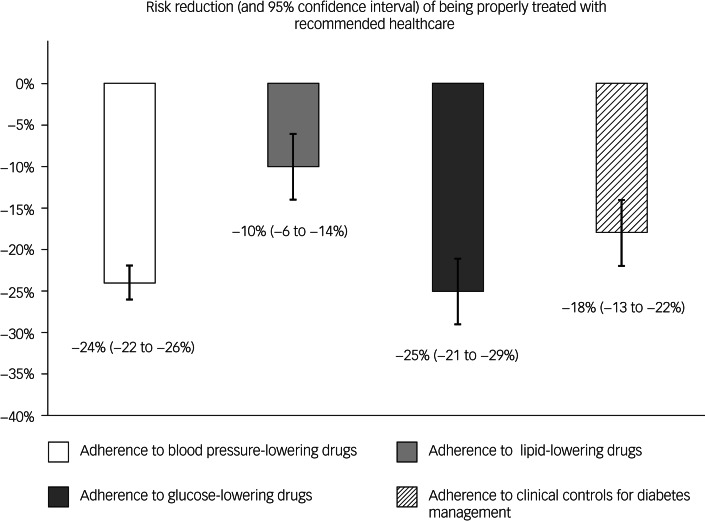
Percentage variation of the likelihood of high adherence to recommendations among patients with severe mental disorder compared with those without evidence of severe mental disorder, and corresponding 95% confidence intervals. Percentage variation of the likelihood of high adherence to recommendations was derived from the quantity (odds ratio−1)×100. The corresponding 95% confidence interval was obtained from the 95% confidence interval of the odds ratio. The latter was estimated with conditional logistic regression. Estimates are adjusted for the covariates listed in Table 1.

As shown in Fig. 2, during the first year of follow-up, cohort members with mental disorders experienced lower adherence to the corresponding specific drug therapy compared with their comparators. With the exception of serum creatinine and dilated eye examination, patients with comorbid diabetes and a severe mental disorder had lower adherence to individual recommendations for clinical controls than the reference cohort (mainly glycated haemoglobin and urine albumin excretion). Study cohort members consistently had significant lower odds of high adherence to drugs and clinical controls than comparators, and the gap between study and reference cohort members was higher for glucose- and blood pressure-lowering agents than for lipid-lowering agents (Fig. 3). Indeed, compared with the reference cohort, patients with a severe mental disorder had reduced likelihood of high adherence to blood pressure-lowering, lipid-lowering or antidiabetic drug therapies of −24% (95% CI −26 to −22%), −10% (95% CI −14 to −6%) and −25% (95% CI −29 to −21%), respectively. A reduced likelihood of high adherence to recommendations for diabetes management of −18% (95% CI −22 to −13%) was also observed among patients with diabetes and severe mental disorder, compared with those with diabetes but no mental disorder.

Stratified analysis showed that type of mental disorder, gender and clinical profile were significant effect modifiers (Table 2). That is, the gap between patients with and without mental disorders was even more pronounced than that obtained from the comparison of whole cohorts, among men (always), among patients affected by personality disorders (usually, except for the adherence to lipid-lowering agents) and among those with good clinical profile (restricted to patients with diabetes). There was no evidence that the adherence gap associated with mental disorders differed among age categories.

**Table 2:** Percentage variation of the likelihood of high adherence to recommendations among patients with severe mental disorder compared with those without a severe mental disorder, according to type of mental disorders, gender, age category and clinical profile, in Lombardy, Italy during 2016–2017

	Prevalent patients treated with:							
	Blood pressure-lowering agents		Lipid-lowering agents		Glucose-lowering agents			
	Drug adherence		Drug adherence		Drug adherence		Clinical controls adherence	
	Odds ratio (95% CI)^a^	*P*-value^b^	Odds ratio (95% CI)^a^	*P*-value^b^	Odds ratio (95% CI)^a^	*P*-value^b^	Odds ratio (95% CI)^a^	*P*-value^b^
Type of mental disorder								
Schizophrenia	−26 (−30 to −22)	0.000	−10 (−18 to −2)	0.353	−23 (−30 to −16)	0.022	−25 (−31 to −18)	0.047
Bipolar disorders	−30 (−35 to −24)		−15 (−3 to −25)		−32 (−61 to −21)		25 (−35 to −13)	
Personality disorders	−35 (−40 to −29)		−18 (−29 to −5)		−38 (−47 to −28)		−26 (−36 to −13)	
Depression	−21 (−23 to −18)		−7 (−13 to −2)		−21 (−27 to −15)		−8 (−15 to −−1)	
Gender								
Men	−27 (−30 to −24)	0.030	−18 (−24 to −13)	0.000	−29 (−34 to −24)	0.030	−15 (−21 to −7)	0.049
Women	−23 (−25 to −20)		−3 (−8 to 3)		−21 (−26 to −16)		−20 (−25 to −7)	
Age (years)								
18–40	−40 (−50 to −29)	0.797	2 (−35 to 59)	0.454	−42 (−58 to −21)	0.179	−23 (−24 to 6)	0.613
41–65	−24 (−27 to −21)		−12 (−18 to −7)		−28 (−33 to −23)		−17 (−23 to −11)	
>65	−24 (−27 to 21)		−8 (−13 to −2)		−21 (−26 to −15)		−17 (−23 to −11)	
Clinical profile^c^								
Good	−25 (−27 to −22)	0.972	−8 (−13 to −3)	0.827	27 (−32 to −21)	0.043	−21 (−26 to −15)	0.039
Intermediate	−30 (−37 to −23)		−5 (−13 to 9)		−19 (−30 to −7)		−14 (0 to −25)	
Poor	−20 (−31 to −7)		−20 (−35 to −2)		−12 (−28 to 6)		8 (−21 to 31)	

a. Percentage variation of the likelihood of high adherence to recommendations was derived from the quantity (odds ratio−1)×100. The corresponding 95% confidence interval was obtained from the 95% confidence interval of the odds ratio. The latter was estimated with conditional logistic regression. Estimates are adjusted for the covariates listed in Table 1.

b. Testing the null hypothesis of homogeneity between-strata (type of mental disorder, gender) or trend along-strata (age, clinical profile).

c. According to the Multisource Comorbidity Score.

The relationship between the presence of a severe mental disorder and high adherence to the recommended healthcare did not substantially change when varying the thresholds for the categorisation of high adherence levels (Supplementary Table 2).

## Discussion

In our study, the 55 162 patients with severe mental illness who were receiving chronic drug therapies had a likelihood of high adherence to blood pressure-lowering, lipid-lowering or antidiabetic agents of −24%, −10% and −25%, respectively, compared with patients without severe mental illness who were receiving the same drugs. Consistently, the 9250 patients with comorbid diabetes and a severe mental disorder had 18% lower likelihood of meeting recommendations for the management of diabetes compared with those with diabetes without a severe mental disorder. The healthcare gap of patients with mental disorders was wider for men and those with personality disorders (although it also affected patients with schizophrenia, bipolar disorder and, to a lesser extent, depression), and had major extension among patients with good clinical profile. These findings should be interpreted in light of the design from which they were generated. Because patients with severe mental disorders were receiving treatment from mental health services, we expect that they had an advantage in terms of medical assistance and healthcare access, which patients without mental disorders did not. This means that the real gap between patients with and without mental disorders is expected to be wider than that observed in our study for individuals with severe mental disorder who do not receive treatment from mental health services.

Our findings are consistent with several meta-analysis and primary studies emphasising treatment gap and unmet needs in psychiatry.^25^ First, acceptability of medications by people living with severe and persistent mental illness has been described as problematic, and adherence to medications is poor.^26^ Second, these issues are often overlooked and/or dismissed by healthcare professionals and policy makers.^27^ For example, physical health concerns of people on antipsychotics were not addressed by prescribing psychiatrists in a UK study.^28^ Finally, the lifespan of persons with serious mental illness has been consistently reported as shortened compared with those without serious mental illness.^29–34^ Up to 50% of their mortality excess is considered potentially preventable through providing timely and high-quality healthcare.^35–40^ Our findings allow for speculation that more careful control of adherence to drug treatments and medical controls, mainly for diabetes and hypertension, would reduce the gap between patients with and without severe mental illness, and perhaps reduce the increased risk of cardiovascular morbidity and mortality among patients with mental disorders.^6^ This implies that the management of the physical health of patients with severe mental disorders needs to be urgently addressed by physicians and decision makers.

The present study is unique in several respects. The investigation is based on routine clinical practice delivered by the Italian National Health System, a free-of-charge universal healthcare system covering essential health needs of all citizens. High-quality interconnectable individual data on out-patient and in-patient services supplied by the NHS, including healthcare provided by the public DMHs, offers the unique opportunity of tracing the complete care pathway of large, unselected populations. The resulting real-world evidence are free from selective participation or recall bias.

Our study has several limitations. Common sources of exposure misclassification include treatments dispensed by private services, as well as out-of-pocket payments. A pitfall of the present study concerns lack of clinical data (e.g. severity of hypertension, hyperlipidaemia and diabetes, other related complications, and comorbidities) as well as socioeconomic information (e.g. economic status and family guardianship), potentially affecting the adherence to chronic drug therapies. Although socioeconomic status can be confidently ruled out because we have previously found that in Lombardy income and educational differences play no role in the persistence on antihypertensive drug treatment,^40^ and despite the fact that the effect of some clinical factors has been carefully controlled in our study by means of stratified analysis, a deeper knowledge of clinical and social traits would have allowed us to more clearly explain some of the findings.

In conclusion, one of the critical barriers to the uptake of mental health programmes is the so-called physical healthcare gap, a concern raised by the relatively frequent but highly unattended physical comorbidity and early mortality of persons with severe mental illness. Our paper showed that patients living with mental illness who needed chronic drug therapy with blood pressure-lowering, lipid-lowering or antidiabetic agents were treated worse than patients without signs of mental disorders but with the same need of chronic drug therapies. The gap in sufficient utilisation of adequate treatment options was particularly pronounced for patients with personality disorders (although it also affected patients with schizophrenia, bipolar disorder and, to a lesser extent, depression), of male gender and with a good general clinical profile.

We hope that the current paper may motivate the research in this field and that further investigations address the causes of non-adherence and effective interventions to overcome the physical healthcare gap of patients with severe mental illness. Meanwhile, individual (from patients, families, psychiatrists, physicians and decision makers) and system-level actions, promoting a joint approach to physical and mental health by mental health professionals and general practitioners, should be considered a priority to adequately address an often ignored problem with a major impact on public health.

## Data Availability

The data that support the findings of this study are available from the Lombardy Region, but restrictions apply to the availability of these data, which were used under license for the current study and so are not publicly available. Data are, however, available from the authors upon reasonable request, and with permission of Lombardy Region.

## Supplementary material

For supplementary material accompanying this paper visit http://dx.doi.org/10.1192/bjo.2021.998. click here to view supplementary material
