# Supplementary material for: Assessing the physical healthcare gap among patients with severe mental illness: large real-world investigation from Italy
Source: BJPsych Open. 2021 Sep 9;7(5):e165. doi: 10.1192/bjo.2021.998 (PMC8444055; doi:10.1192/bjo.2021.998)
Supplement: Supplementary file 1 [file S2056472421009984sup001.docx]

**Assessing physical healthcare gap among patients with severe mental illness: a large real-world investigation from Italy**

Giovanni CORRAO^1,2^, Matteo MONZIO COMPAGNONI^1,2^, Valeria VALSASSINA^1,2^, Antonio LORA^1,3^

^1^ National Centre for Healthcare Research and Pharmacoepidemiology, University of Milano-Bicocca, Milan, Italy

^2^ Unit of Biostatistics, Epidemiology and Public Health, Department of Statistics and Quantitative Methods, University of Milano-Bicocca, Milan, Italy

^3^ Department of Mental Health and Addiction Services, ASST Lecco, Lecco, Italy

**SUPPLEMENTARY MATERIAL**

**Address for correspondence**: Dr. Matteo Monzio Compagnoni, Department of Statistics and Quantitative Methods, Division of Biostatistics, Epidemiology and Public Health, University of Milano-Bicocca, Street Bicocca degli Arcimboldi, 8, Building U7, 20126 Milan, Italy. Phone: +39.02.64485859; E-mail: [matteo.monziocompagnoni@unimib.it](mailto:matteo.monziocompagnoni@unimib.it)

**Table S1**. Diagnostic and therapeutic (ICD-9-CM, ICD-10, and ATC) codes used in the current study for drawing records and fields from Healthcare Utilization databases.

| **DEPRESSION** | |
| --- | --- |
|  | **ICD-10 codes** |
| Depressive episode | F32.* |
| Recurrent depressive disorder | F33.* |
| Dysthymia | F34.1 |
| Other persistent mood [affective] disorders | F34.8 |
| Persistent mood [affective] disorder, unspecified | F34.9 |
| Other recurrent mood [affective] disorders | F38.1 |
| Other specified mood [affective] disorders | F38.8 |
| Unspecified mood [affective] disorder | F39.* |
| Post-traumatic stress disorder | F43.1 |
| Adjustment disorders | F43.2 |
| **SCHIZOPHRENIA** | |
|  | **ICD-10 codes** |
| Schizophrenia | F20.* |
| Schizotypal disorder | F21.* |
| Delusional disorders | F22.* |
| Brief psychotic disorder | F23.* |
| Shared psychotic disorder | F24.* |
| Schizoaffective disorders | F25.* |
| Other psychotic disorder not due to a substance or known physiological condition | F28.* |
| Unspecified psychosis not due to a substance or known physiological condition | F29.* |
| **BIPOLAR DISORDER** | |
|  | **ICD-10 codes** |
| Manic episode | F30.* |
| Bipolar affective disorder | F31.* |
| Cyclothymia | F34.0 |
| Other single mood [affective] disorders | F38.0 |
| **PERSONALITY DISORDER** | |
|  | **ICD-10 codes** |
| Specific personality disorders | F60.* |
| Mixed and other personality disorders | F61.* |
| **Other diseases** | |
|  | **ICD-9-CM codes** |
| Cancer | 140.x – 208.x |
| Diabetes | 250.x |
| **DRUGS** | |
|  | **ATC codes** |
| Lipid-lowering agents | C10AA |
| Blood pressure-lowering agents | C02, C03, C07, C08, C09 |
| Antidiabetic agents | A10 |
| Drugs for COPD | R03 |
| NSAIDs | M01A, M02A |
| Digitalis | C01AA |
| Nitrates | C01DA |
| Antithrombotic agents | B01 |
| Antiarrhythmics | C01B |
| Antineoplastic drugs | L |
| **OUTPATIENT PROCEDURES** | |
|  | **Regional procedure codes** |
| Glycated haemoglobin | 90.27.1, 90.28.1 |
| Lipid profile | 90.14.1, 90.14.3, 90.43.2 |
| Serum creatinine | 90.16.3, 90.16.4 |
| Urine albumin excretion | 90.33.4 |
| Dilated eye exam | 95.02; 95.09.1 |
|  |  |

**Table S2**. Percentage variation of the likelihood to be well adherent to recommendations among patients with severe mental disorder with respect to those without evidence of severe mental disorder, and corresponding 95% CI; data are shown according with different criteria/thresholds for defining good adherence.

|  | Proportion of days covered by drug availability beyond the which good adherence was assumed | | |
| --- | --- | --- | --- |
| Prevalent users of… | **≥ 60%** | **≥ 80%** | **≥ 90%** |
| blood pressure-lowering agents | -27%  (-25% to -29%) | -24%  (-22% to -26%) | -20%  (-18% to 22%) |
| lipid-lowering agents | -8%  (-4 to -11%) | -11%  (-7% to -15%) | -12%  (-7% to -16%) |
| glucose-lowering agents | -28%  (-24% to -31%) | -23%  (-19% to -27%) | -20%  (-16% to -24%) |

Percentage variation of the likelihood to be well adherent to recommendations was derived from the quantity (odds ratio - 1)*100. The corresponding 95% confidence interval was obtained from the 95% confidence interval of odds ratio. The latter was estimated with conditional logistic regression. Estimates are adjusted for the covariates listed in Table 1.
